# Flight muscle size reductions and functional changes following long‐distance flight under variable humidity conditions in a migratory warbler

**DOI:** 10.14814/phy2.15842

**Published:** 2023-10-17

**Authors:** Derrick J. E. Groom, Betsy Black, Jessica E. Deakin, Joely G. DeSimone, M. Collette Lauzau, Bradley P. Pedro, Chad R. Straight, Kimberly P. Unger, Mark S. Miller, Alexander R. Gerson

**Affiliations:** ^1^ Department of Biology University of Massachusetts Amherst Massachusetts USA; ^2^ Department of Biology San Francisco State University California San Francisco USA; ^3^ Centre for Animals on the Move, Department of Biology Western University Ontario London Canada; ^4^ Department of Kinesiology University of Massachusetts Massachusetts Amherst USA; ^5^ Present address: Center for Ecosystem Science and Society Northern Arizona University Arizona Flagstaff USA; ^6^ Present address: Appalachian Laboratory University of Maryland Center for Environmental Science Maryland Frostburg USA; ^7^ Present address: The Water School Florida Gulf Coast University Florida Fort Myers USA; ^8^ Present address: Department of Biology Tufts University Massachusetts Medford USA

**Keywords:** atrophy, dehydration, exercise, migration, skeletal muscle

## Abstract

Bird flight muscle can lose as much as 20% of its mass during a migratory flight due to protein catabolism, and catabolism can be further exacerbated under dehydrating conditions. However, the functional consequences of exercise and environment induced protein catabolism on muscle has not been examined. We hypothesized that prolonged flight would cause a decline in muscle mass, aerobic capacity, and contractile performance. This decline would be heightened for birds placed under dehydrating environmental conditions, which typically increases lean mass losses. Yellow‐rumped warblers (*Setophaga coronata*) were exposed to dry or humid (12 or 80% relative humidity at 18°C) conditions for up to 6 h while at rest or undergoing flight. The pectoralis muscle was sampled after flight/rest or after 24 h of recovery, and contractile properties and enzymatic capacity for aerobic metabolism was measured. There was no change in lipid catabolism or force generation of the muscle due to flight or humidity, despite reductions in pectoralis dry mass immediately post‐flight. However, there was a slowing of myosin–actin crossbridge kinetics under dry compared to humid conditions. Aerobic and contractile function is largely preserved after 6 h of exercise, suggesting that migratory birds preserve energy pathways and function in the muscle.

## 1 INTRODUCTION

In preparation for migration, birds accumulate large fat stores to support the immense energetic demands of migratory flight (Guglielmo, 2010, 2018; Guglielmo et al., 2002; McFarlan et al., 2009; Price et al., 2010). Some organs, such as the flight muscles, heart, and lungs, also undergo significant hypertrophy, substantially increasing in size in preparation for migration (Piersma, 1998). This seasonal organ flexibility is associated with increased functional capacity to support the large metabolic and mechanical costs of migratory flight (Piersma & Lindström, 1997). These organs are then catabolized during long flights, accounting for up to 30% of the total energy expenditure (Dick & Guglielmo, 2019a, 2019b; Gerson & Guglielmo, 2011, 2013; Groom et al., 2019; Guglielmo et al., 2017). As there is no storage form of protein analogous to adipose fat, protein catabolism in flight leads to substantial reductions in organ mass (Battley et al., 2000; Bauchinger et al., 2005; Bauchinger & Biebach, 2001; Biebach & Bauchinger, 2003). Although the causes of protein catabolism are still being elucidated and many hypotheses have been proposed (Bauchinger & Biebach, 1998; Bauchinger & McWilliams, 2010; Gerson & Guglielmo, 2011; Groom et al., 2019; Jenni & Jenni‐Eiermann, 1998), the physiological consequences of protein breakdown in flight have received limited attention. If increases in organ size augment physiological function in preparation for migration, then we predict that the corresponding atrophy and degeneration of these tissues will reduce function. Previous work has shown this to be the case for the digestive tract, as it can face upward of 50% reductions in size following a migratory flight (Battley et al., 2000; Bauchinger et al., 2005; Karasov & Pinshow, 1998), leading to large increases in the mean retention time and changes in nutrient uptake rates until the digestive tract recovers (Bauchinger et al., 2009; Tracy et al., 2010). However, functional costs due to flight atrophy have not been explored in other organ systems of migrating birds and of particular interest is the rapid atrophy of the flight muscle.

The primary flight muscles (the pectoralis and supracoracoideus) show significant flexibility in size over the course of the migratory season. In preparation for migration, the mass of the flight muscles can increase by 35% relative to the winter season (Marsh, 1984). Much of this increase is due to hypertrophy of the myofibrils and increases in mitochondrial density, as ultrastructural investigations have shown increases in muscle area and mitochondrial content (Evans et al., 1992). However, after a migratory flight the pectoralis muscle mass can be reduced by up to ~26% of initial predeparture mass (Battley et al., 2000), indicating that the muscle is actively catabolized during flight. Additionally, histological examination of myofibrils following migration show Z‐line streaming, disrupted mitochondria, lipofuscin (George et al., 1987), and an increase in plasma creatine kinase (Guglielmo et al., 2001), all evidence of exercise‐induced muscle damage and atrophy. Other studies in the trans‐Saharan migrant the garden warbler (*Sylvia borin*) have demonstrated that the protein losses in muscle are primarily in the myofibrillar elements, with relative sparing of sarcoplasmic proteins (Bauchinger & Biebach, 2001). Overall, this suggests that the largest changes in mass are due to the loss of contractile elements relative to all muscle proteins, raising the prospect that muscle atrophy could reduce contractile function.

In addition to the effect of exercise, the environmental conditions experienced during flight can also have a profound impact on the amount of lean mass a bird catabolizes. More dehydrating conditions elicit greater amounts of lean mass loss across the entire animal (Gerson & Guglielmo, 2011; Groom et al., 2019), suggesting that increased protein catabolism may be a component of the environmental stress response. As protein catabolism generates more metabolic water per kJ of energy than other fuel sources (Giulivi & Ramsey, 2015; Jenni & Jenni‐Eiermann, 1998), selective breakdown protein may counterbalance the heightened rate of water loss in dry environmental conditions (Gerson & Guglielmo, 2011). Thus, there is likely a basal level of protein catabolism of the flight muscles occurring during migration, which can then be heightened by environmental stress (Groom et al., 2019). Whether the mechanisms or functional consequences of stress‐induced protein catabolism differs from that of exercise‐induced “basal” levels of breakdown is currently unknown. Given the high degree of heterogeneity in where the organ size losses occur (Battley et al., 2000), the lean mass losses induced by environmental stress may not occur at the flight muscle at all, and the muscle could be spared from additional dehydration‐related catabolism. On the other hand, the flight muscles make up a substantial proportion of the bird's mass (up to 35% [Greenewalt, 1975]) making them the largest repository of protein. Environmental stressors may increase the amount of protein that is mobilized from the muscle, and thus exacerbate the rate of muscle loss during a migratory flight.

We flew yellow‐rumped warblers (*Setophaga coronata*) in a temperature‐ and humidity‐controlled wind tunnel for upward of 6‐h to determine the changes in muscle mass, metabolic capacity, and the contractile function that may occur due to long‐duration flight. We hypothesized that pectoralis muscle size will decline as lean mass declines during flight, with this loss further exacerbated by high evaporative water loss (i.e., dehydrating) conditions, as found previously (Gerson & Guglielmo, 2011; Groom et al., 2019). We also predicted that the declines in size would be mirrored by declines in muscular function, including reductions in the mass‐specific metabolic capacity and single‐fiber contractile performance, regardless of whether the loss was exercise‐induced or caused by environmental stress, leading to overall greater losses of muscle mass and function for birds flown under dehydrating conditions.

## 2 METHODS

### 2.1 Animals

Yellow‐rumped warblers (*Setophaga coronata*) were captured near Long Point Bird Observatory, Ontario, Canada in October 2016 and 2017 by mist netting. Birds were immediately transported to the Advanced Facility for Avian Research at Western University, London, ON, Canada, and morphometrics were taken upon arrival (the length of the exposed culmen, nares to the tip of the bill, tarsus, sternum length, wing chord, and the width of the bill). Each bird was banded with a unique color band combination for identification. Birds were either housed in indoor aviaries (3.7 m × 2.1 m × 3.0 m) or in individual smooth‐sided cages (121 cm × 68 cm × 185 cm) at approximately 20°C and on a 12L:12D photoperiod. Birds were provided with synthetic diet (see (Groom et al., 2019) for diet composition) and water ad libitum, supplemented with 10 *Tenebrio molitor* larvae per bird daily. Birds from both years were used in a separate flight study prior to the beginning of this study; birds were allowed to recover for at least 2 weeks before commencing flights. All animal procedures and care followed Canadian Council on Animal Care guidelines and were approved by Western University Animal Care Committee (protocol 2010‐216) and the University of Massachusetts Amherst Institutional Animal Care and Usage Committee (protocol 2015‐0019). Permission for animal capture was provided by the Canadian Wildlife Service (permit CA‐0256 to Dr. Christopher Guglielmo).

### 2.2 Wind tunnel flights

As these birds are nocturnal migrants, all flights occurred at night. Birds were fasted for one hour before the beginning of flight. After lights off, birds were removed from their cages and weighed to the nearest 0.001 g. Body composition (fat and fat‐free mass) was determined by quantitative magnetic resonance (QMR, Echo‐MRI) calibrated to a 15.0 g canola oil standard and set to three accumulations. Immediately after scanning, birds were flown individually at 8.0 m s^−1^ at 18°C in either low evaporative water loss (LEWL; 12 g water m^−3^, equivalent to 80% relative humidity) or high evaporative water loss (HEWL; 2 g water m^−3^, equivalent to 12% relative humidity) conditions. Flights were ended when the bird ceased flying three times within 5 min, or at 6 h (Dick & Guglielmo, 2019a, 2019b). Each flying bird was paired with a time‐matched resting bird that experienced the same handling, QMR protocol, and environmental conditions as the flight bird but did not fly in the wind tunnel. Instead, the rest bird was placed in a covered cage within the plenum of the wind tunnel. At the end of the flight, both the rest and flight birds were immediately weighed, scanned again using the QMR, and blood sampled via brachial puncture (~70 μL). Birds were then immediately euthanized by an overdose of inhalant isoflurane followed by cervical dislocation or decapitation and quantitatively dissected (see below). There was a slight delay in sampling and data collection between the flight and rest bird. The flight bird was immediately processed at the end of flight while the rest bird remained within the wind tunnel under the prescribed humidity treatment. Upon completing the sampling procedures with the flight bird, the rest bird was removed from the wind tunnel for sampling. The time of removal of the rest bird from the wind tunnel was recorded as the end of the rest period. This sequence was selected so that a single individual performed all dissections to reduce variability among experimenters. A subset of the yellow‐rumped warblers from 2017 were allowed to recover following flight/rest in the wind tunnel after their second QMR scan. During recovery, birds had ad libitum access to water and the regular diet (described above). Twenty‐four hours after the beginning of the experimental flight/rest in the wind tunnel, the recovery birds were QMR scanned a third time before tissue and blood sampling. A set of “preflight control” birds for each year were prepared for flight in the same manner as the experimental birds but were euthanized and sampled before entry into the wind tunnel.

Changes in total body mass, fat mass, and fat‐free mass were calculated as the difference between pre‐ and post‐flight/rest QMR scans. The energetic requirement for flight was calculated using the change in fat and fat‐free mass, assuming an energy density of 37.6 kJ g^−1^ and 5.3 kJ g^−1^ for fat and fat‐free mass, respectively (Jenni & Jenni‐Eiermann, 1998).

### 2.3 Dissections

The left pectoralis was excised and weighed on a precision balance within 5 min of euthanasia. A small portion of the pectoralis (~0.0100 g) was weighed, dried at 60°C for 48 h, and weighed again to measure the percent water content of the tissues. The remaining left pectoralis and the entire right pectoralis was immediately frozen in liquid nitrogen and then stored at −80°C. Dry pectoralis mass was calculated as the wet pectoralis mass multiplied by the percent dry mass. In 2017, a subsection of the right pectoralis was removed before being frozen in liquid nitrogen and prepared for measurement of contractile properties as described below. Measurements of contractile properties were only performed on birds sampled immediately after flight or resting in the wind tunnel.

### 2.4 Muscle fiber contractile properties

Pectoralis muscle fibers were dissected into individual muscle bundles and placed in skinning solution (170 mM potassium propionate, 10 mM imidazole, 5 mM EGTA, 2.5 mM MgCl_2_, 2.5 mM ATP, and a Complete Minitab protease inhibitor, pH 7.0) for ~12 h at 4°C. After initial skinning, fibers were placed in a modified skinning solution (addition of 1.00 mM sodium azide, but no protease inhibitors) with increasing glycerol concentration (10% v/v glycerol, 25% v/v glycerol, and 50% v/v glycerol), for 2 h in each solution. After 2 h in the final 50% v/v glycerol‐skinning solution, samples were stored at −20°C. The high concentration of glycerol permeabilize the muscle fiber membrane and prevents the solution from freezing and damaging the fibers (Momb et al., 2022). Samples were transported to the University of Massachusetts Amherst for contractile function measurements.

Preparation of single fibers for mechanical measurements, the testing apparatus, and all experimental solutions have been previously described (Momb et al., 2022). Briefly, on the day of an experiment, individual fibers (~1 mm in length) were isolated from the pectoralis muscle in dissection solution at 4°C, aluminum T‐clips were affixed to both ends, and fiber height‐to‐width ratio was determined by performing top and side diameter measurements. The fiber was demembranated for 30 min in skinning solution (plus 1% v/v Triton X‐100) at 4°C to ensure complete removal of the sarcolemma and sarcoplasmic reticulum. Afterward, the fiber was mounted on a custom‐built testing rig by placing the T‐clips onto hooks attached to a piezo actuator linear motor (P‐841.10, Physik Instrumente, Auburn, MA) and an Akers strain gauge (AE‐801, SensorOne, Sausalito, CA). While in relaxing solution, sarcomere length was set to 2.65 μm and cross‐sectional area (CSA) was determined using an inverted microscope (Zeiss Invertiscope) with a video camera (BFLY‐U3‐23S6m‐C, Point Grey Research, Richmond) and custom video analysis software (ImageJ) (Schneider et al., 2012). Prior to activation at 25°C, the fiber was slackened completely, the force gauge zeroed, the fiber pulled back to its original length, allowed to equilibrate for 1 min, and transferred to pre‐activating solution for 30 s. The fiber was then placed in maximal Ca^2+^‐activated solution, force was measured after it plateaued, sinusoidal analysis was performed, and force was measured again.

Myofilament properties and myosin–actin crossbridge kinetics were derived via sinusoidal analysis, which yields three characteristic processes, A, B and C, which relate to various mechanical (*A*, *B*, *C* and *k*) and kinetic (2π*b* and 2π*c*) properties of the crossbridge cycle, as previously described (Momb et al., 2022). The A‐process (*A* and *k*) under Ca^2+^‐activated conditions reflects the myofilament lattice stiffness and attached myosin heads in series (Mulieri et al., 2002). The B‐ and C‐process magnitudes (*B* and *C*) are proportional to the number of strongly bound myosin–actin heads and/or the stiffness of the crossbridges (Palmer et al., 2007). The frequency portion of the B‐process (2π*b*) is interpreted as the rate of myosin transition from the weakly to strongly bound state, or the (apparent) rate of myosin force production (Zhao & Kawai, 1993). The frequency portion of the C‐process (2π*c*) represents the crossbridge detachment rate, meaning the inverse (2π*c*)^−1^ is the mean myosin attachment time to actin, *t*_on_ (Palmer et al., 2007). Single fiber mechanical experiments were completed within 3 weeks of initial dissection. As small birds demonstrate homogeneity in the fiber type of the pectoralis (Velten & Welch, 2014; Welch Jr. & Altshuler, 2009), we assumed that all fibers were fast oxidative‐glycolytic fibers.

### 2.5 Enzyme and biochemical assays

Frozen pectoralis muscle was homogenized as described in Price et al. (Price et al., 2010). Tissue samples from both years were homogenized and assayed at the same. Briefly, approximately 50–100 mg of pectoralis tissue was homogenized in nine volumes of homogenization buffer (20 mM Na_2_HPO_4_, 0.5 mM EDTA, 0.2% w/v bovine serum albumin, 50% v/v glycerol, 0.1% v/v Triton X‐100, pH 7.4) using a Bullet Blender (Next Advance, Inc) for 5 min at 4°C. All homogenization occurred on a different day than the enzyme assays, and homogenized samples were stored at −80°C until assayed. The homogenization buffer stabilizes the enzymes during freezing (Hochachka & Mommsen, 1994).

Assays for lactate dehydrogenase (LDH), citrate synthase (CS), 3‐hydroxyacyl‐CoA dehydrogenase (HOAD), and carnitine palmitoyl transferase (CPT) were measured as described in Price et al. (Price et al., 2010). Alanine aminotransferase (ALT) was measured according to Bergmeyer et al. (Bergmeyer et al., 1978). All assays were performed at 39°C in a 96‐well plate reader (Synergy H‐1, Biotek, Winooski VT, USA). Protein content was measured using Bradford's reagent, using bovine serum albumin as a standard. Enzyme activities are expressed as mass‐specific (μmol min^−1^ g wet tissue^−1^).

Plasma creatine kinase was measured using a Creatine Kinase kit (Sigma‐Aldrich). Plasma was diluted three‐fold using 0.9% saline solution before analysis.

### 2.6 Statistics

All analyses were performed using R v3.3.0 (2019), and ANCOVAs were performed using the car package (v. 3.0‐3). An alpha of 0.05 was used throughout. Model assumptions were checked visually using residual and quantile‐quantile plots.

Initial body composition (body mass, fat mass, and fat‐free mass) across the experimental groups was analyzed using one‐way ANCOVAs. As preflight controls cannot be assigned a time point, humidity treatment, or flight treatment, the treatments for the experimental groups were collapsed into a single factor to allow for valid comparisons to the preflight control group. The first principal component was included as a covariate to account for differences in structural body size (Rising & Somers, 1989).

Changes to body composition (body mass, fat mass, and fat‐free mass) when flying or resting in the wind tunnel were analyzed using general linear models, and included flight treatment (flight/rest), humidity treatment (HEWL/LEWL), and duration in wind tunnel. As the preflight controls were not flown, they were excluded from this analysis.

Wet and dry pectoralis mass was analyzed using general linear models, and included duration in the wind tunnel, humidity, flight treatment, sampling time point (sampled either immediately upon the conclusion of the experimental flight/rest in the wind tunnel or after recovery, hereby known as “post” and “recovery” groups, respectively), wing chord, and year as factors. Nine birds were excluded from this analysis due to incomplete pectoralis dissections, as assessed by a follow‐up examination of frozen carcasses. Because the preflight controls cannot be assigned a flight treatment, humidity treatment, or a time point, they were excluded from this analysis.

To make valid comparisons between preflight controls and the experimental groups, wet and dry pectoralis mass of preflight control birds were compared with birds with wind tunnel durations of 5 h or longer. Flight treatment and sampling time point were concatenated into a single factor, and wing chord was used as a covariate to account for differences in body size, and a one‐way ANCOVA was performed with a post hoc Tukey's honestly significant differences test. Because humidity treatment was not found to be a significant factor on wet or dry pectoralis mass (see Results), humidity was not included as a factor for this analysis.

Enzyme and protein content data of the birds placed in the wind tunnel were analyzed using ANCOVA. Flight treatment, humidity treatment, time point, and duration in the wind tunnel were included as factors. Because long‐term storage of tissues may have an impact on maximum enzymatic rates, the effect of year was assessed in the 2016 and 2017 preflight control birds using Welch's two‐sample *t*‐tests on the preflight controls. If the *t*‐test found a significant difference between the 2 years for a particular enzyme, year was also included in the enzyme models as an additional factor.

Following the previous analysis, duration in the wind tunnel and humidity treatment were not significant factors. The enzyme activities and protein content of all the experimental birds were compared with the preflight control birds by concatenating flight treatment and time point as a single factor, followed by a one‐way ANOVA and post hoc analysis using Tukey's honestly significant differences test.

Contractile properties were analyzed using linear‐mixed effects models, including humidity and flight treatment as factors and Bird ID was used as a random effect.

Sex of the birds was initially included as a covariate for all of the models, but was not a significant factor across any of the analyses. Thus, sex was removed from the models and the models were rerun.

## 3 RESULTS

### 3.1 Initial body condition

A total of 80 individuals were tested (preflight control: 16; HEWL‐postflight: 9; LEWL‐postflight: 10; HEWL‐post‐rest: 9; LEWL‐post‐rest: 10; HEWL‐recovery‐flight: 7; LEWL‐recovery‐flight: 6; HEWL‐recovery‐rest: 7; LEWL‐recovery‐rest: 6). Although treatment groups were selected randomly, initial body mass was significantly different between treatment groups (total treatment: *F*_8,70_ = 3.155, *p* = 0.004, PC1: *F*_1,70_ = 5.193, *p* = 0.026; Table 1). The effect of total treatment was driven entirely by the LEWL‐recovery‐rest groups being lower in body mass than HEWL‐postflight, LEWL‐postflight, and LEWL‐recovery‐flight groups (Table 1). However, this variation was due to initial fat, as fat was significantly different between LEWL‐recovery‐rest and HEWL‐postflight group and bordering on significance between LEWL‐recovery‐rest and LEWL‐postflight (treatment: *F*_8,70_ = 2.24, *p* = 0.035; PCA1: *F*_1,70_ = 0.51, *p* = 0.48). There was no difference in initial fat‐free mass across all of the groups, after accounting for body size (treatment: *F*_8,70_ = 1.496, *p* = 0.175; PC1: *F*_1,70_ = 30.976, *p* < 0.001; Table 1).

**TABLE 1:** Initial mean whole‐animal, fat, and fat‐free masses across each experimental group upon entry into the experiment.

Treatment	Humidity	Time point	Whole‐animal mass (g)	Fat mass (g)	Fat‐free mass (g)
Preflight control			12.70 ± 1.06	1.89 ± 1.04	10.81 ± 0.77
Flight	HEWL	Post	13.66 ± 0.73^b^	2.62 ± 0.95^y^	11.04 ± 0.70
		Recovery	12.76 ± 0.76	1.75 ± 0.53	11.01 ± 0.59
	LEWL	Post	13.55 ± 1.39^b^	2.43 ± 0.91	11.12 ± 0.98
		Recovery	13.27 ± 1.19	2.22 ± 0.94	11.05 ± 0.41
Rest	HEWL	Post	12.98 ± 1.59	2.07 ± 1.42	10.90 ± 0.55
		Recovery	12.46 ± 0.67	1.36 ± 0.72	11.10 ± 0.62
	LEWL	Post	12.60 ± 1.16	1.77 ± 1.02	10.82 ± 0.51
		Recovery	11.04 ± 0.99^a^	0.90 ± 0.66^z^	10.15 ± 0.55

*Note*: Different letters within a column indicate significantly different initial masses in that category. Values are means ± SD.

### 3.2 Wind tunnel body composition changes and energetics

**FIGURE 1:**
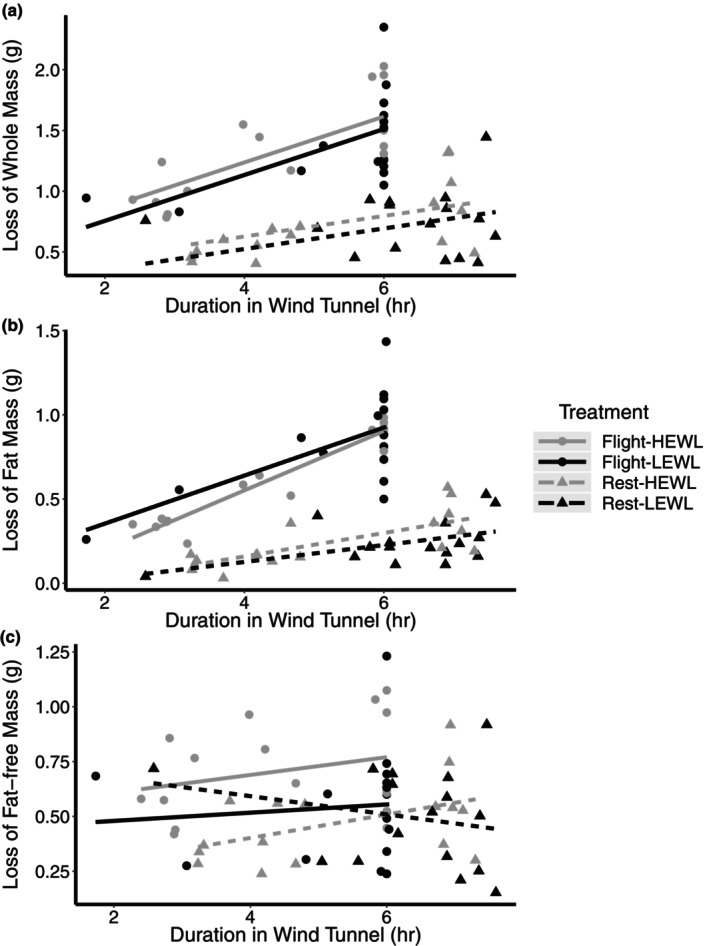
The effect prolonged flight or rest under HEWL and LEWL conditions on the loss of (a) whole‐animal, (b) fat, and (c) nonfat mass. HEWL birds are represented by gray and LEWL in black. Flight birds are represented by circles and solid lines, and rest birds by triangles and dashed lines. There was a significant effect of flight treatment and duration in the wind tunnel on whole‐animal mass and fat mass. There was a significant effect of humidity and flight treatment on fat‐free mass loss. Sample sizes of each experimental group: Flight/HEWL/Recovery = 7; Flight/LEWL/Recovery = 6; Rest/HEWL/Recovery = 7, Rest/LEWL/Recovery = 6; Flight/HEWL/Post = 9; Flight/LEWL/Post = 10; Rest/HEWL/Post = 9; Rest/LEWL/Post = 10.

Flight birds lost mass at a significantly higher rate than rest birds (flight treatment x duration in the wind tunnel: *F*_1,59_ = 4.743, *p* = 0.033; flight treatment: *F*_1,59_ = 0.520, *p* = 0.484; duration in the wind tunnel: *F*_1,59_ = 27.718, *p* < 0.001; Figure 1a), with flight birds losing mass at 0.189 ± 0.035 g hr^−1^ (95% CI: 0.062–0.163 g hr^−1^) compared to the rest birds 0.085 ± 0.034 g hr^−1^ (95% CI: 0.016–0.153). Humidity did not have a significant effect on total mass loss (*F*_1,59_ = 1.942, *p* = 0.169).

Fat mass was lost at a significantly higher rate in flight birds than rest birds, with flight birds losing fat mass at 0.169 ± 0.018 g hr^−1^ (95% CI: 0.131–0.207) and rest birds at 0.046 ± 0.015 g hr^−1^ (95% CI: 0.016–0.077) (flight treatment x duration in wind tunnel: *F*_1,59_ = 20.726, *p* < 0.001; flight treatment: *F*_1,59_ = 0.038, *p* = 0.846; duration in wind tunnel: *F*_1,59_ = 80.660, *p* < 0.001; Figure 1b). There was no effect of humidity on fat loss (*F*_1,59_ = 0.041, *p* = 0.840).

Flight birds lost 0.600 ± 0.106 g fat‐free mass compared to 0.342 ± 0.120 g lost by the rest birds (*F*_1,59_ = 10.458, *p* = 0.002; Figure 1c). Additionally, birds under the HEWL conditions lost 0.188 ± 0.080 g more fat‐free mass than LEWL birds (*F*_1,59_ = 5.465, *p* = 0.023). Duration in the wind tunnel was not a significant factor (*p* > 0.1).

Flight costs increased with longer duration in the wind tunnel, with flight birds consuming energy at 6.474 ± 0.737 kJ hr^−1^ and rest birds at 2.219 ± 0.700 kJ hr^−1^ (flight treatment x duration in wind tunnel: *F*_1,59_ = 77.152, *p* < 0.001; flight treatment: *F*_1,59_ = 0.001, *p* = 0.980; duration in wind tunnel: *F*_1,59_ = 18.704, *p* < 0.001). Humidity was not a significant factor for energy usage (*F*_1,59_ = 0.279, *p* = 0.560).

### 3.3 Recovery of body condition

**FIGURE 2:**
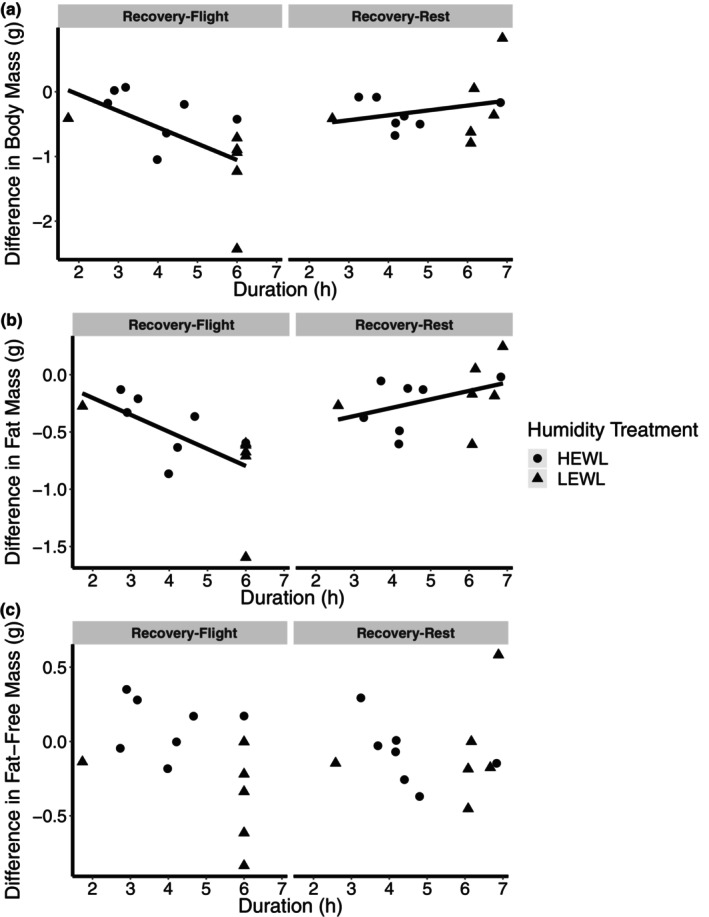
Difference in (a) whole‐animal, (b) fat mass, and (c) fat‐free mass between preexperimental and recovery of flight and rest birds, in relation to duration spent in the wind tunnel. There was a significant effect of duration in the wind tunnel and flight treatment on fat and whole‐animal mass. Humidity was not a significant factor. (sample size: Flight‐HEWL = 7; Flight‐LEWL = 6; Rest‐HEWL =. 7; Rest‐LEWL = 6).

The recovery of body condition in the recovery group of birds was performed by calculating the difference between recovery and preexperimental condition. After controlling for structural body size using PCA1 (*F*_1,20_ = 0.81, *p* = 0.38; Figure 2a), there was a significant effect of flight treatment and wind tunnel duration on the recovery of whole‐animal body mass (flight treatment: *F*_1,20_ = 2.44, *p* = 0.13; duration in the wind tunnel: *F*_1,20_ = 5.47, *p* = 0.030; duration in the wind tunnel x flight treatment: *F*_1,20_ = 5.62, *p* = 0.028; Figure 2a), with longer flight durations associated with greater body mass deficits relative to the initial body mass. Rest birds, however, recovered nearly all of their whole‐animal body mass, regardless of their duration. Humidity was not a significant factor (*F*_1,20_ = 0.50, *p* = 0.49).

Similar to whole‐animal body mass, flight treatment and wind tunnel duration had a significant effect on fat mass recovery (duration in the wind tunnel: *F*_1,20_ = 7.24, *p* = 0.014; flight treatment: *F*_1,20_ = 2.54, *p* = 0.13; duration in the wind tunnel x flight treatment: *F*_1,20_ = 2.80, *p* = 0.011; Figure 2b), with longer duration flight birds having greater fat deficits relative to initial condition after controlling for wing chord (*F*_1,20_ = 1.10, *p* = 0.28). The rest birds had greater recovery of fat over the recovery period. Humidity was not a significant factor (*F*_1,20_ = 0.00, *p* = 0.99).

Recovery of fat‐free mass was not significantly different across the different treatment groups or across durations (*p* > 0.1; Figure 2c).

### 3.4 Plasma creatine kinase

There was no significant effect of humidity treatment or duration in the wind tunnel on plasma creatine kinase concentration (humidity: *F*_1,21_ = 0.128, *p* = 0.724; duration in the wind tunnel: *F*_1,21_ = 0.669, *p* = 0.423), so humidity and duration were dropped from the analysis comparing among all flight treatment groups (control, rest, and flight). There was a significant difference between flight treatment groups (*F*_2,32_ = 11.108, *p* < 0.001). Post hoc testing revealed that flight birds had the highest plasma creatine kinase activity among all groups (1.538 ± 0.600 U/L), around 3.5 times higher than control birds (0.440 ± 0.195 U/L) and 50% higher than rest birds (0.955 ± 0.661 U/L). Rest and control birds were not significantly different.

### 3.5 Wet and dry pectoralis muscle mass

**FIGURE 3:**
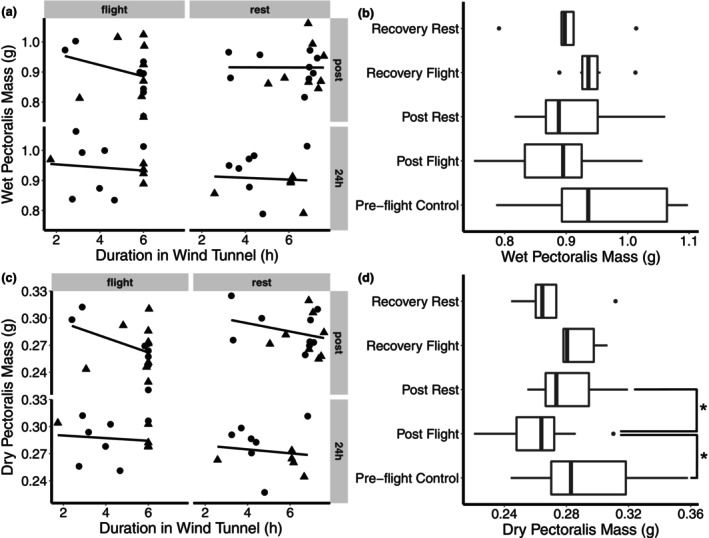
Pectoralis (a) wet and (c) dry mass in relation to duration in the wind tunnel of flight and rest birds. Birds were either sampled immediately after experimental treatment or 24 h after the beginning of the experimental treatment. There was a significant interaction between flight treatment and duration in the wind tunnel on dry pectoralis mass. Pectoralis (b) wet and (d) dry mass of preflight controls were compared with experimental flight and rest birds with durations greater than 5 h. Humidity was not a significant factor and was subsequently removed in this latter analysis. *p < 0.05. (Sample sizes for figures A and C: Flight/HEWL/Recovery = 7; Flight/LEWL/Recovery = 6; Rest/HEWL/Recovery = 7, Rest/LEWL/Recovery = 5; Flight/HEWL/Post = 8; Flight/LEWL/Post = 9; Rest/HEWL/Post = 9; Rest/LEWL/Post = 8; Preflight control = 13. Sample sizes for figures B and D: Flight/Recovery = 6; Rest/Recovery = 5; Flight/Post = 13; Rest/Post = 14; Preflight control = 13. The number of males, females, and unknown are 38, 39, and 3, respectively, and both sexes were represented in all experimental groups).

There was no effect of humidity treatment on wet pectoralis muscle mass (*F*_1,52_ = 0.0082, *p* = 0.928), and humidity treatment was subsequently dropped from the final linear model. There was a significant effect of time point (*F*_1,53_ = 4.27, *p* = 0.044; Figure 3a), with the wet pectoralis of recovery birds being ~32 mg heavier than the post birds, after accounting for the variation due to body size (wing chord; *F*_1,53_ = 50.00, *p* < 0.001). There was a significant interaction between flight treatment and duration in the wind tunnel (flight treatment x duration: *F*_1,53_ = 5.97, *p* = 0.018; flight treatment: *F*_1,53_ = 3.92, *p* = 0.053; duration *F*_1,53_ = 2.52, *p* = 0.12), with flight birds with longer durations in the wind tunnel having lower pectoralis wet mass.

The wet pectoralis mass of the preflight control birds was compared with flight and rest birds with durations in the wind tunnel greater than or equal to 5 h. Because humidity was not a significant factor in the previous model, it was not included as a factor here. Time point (post/recovery/control) and treatment (flight/rest/control) were concatenated into a single factor for analysis using a one‐way ANCOVA. Only wing chord was significant (*F*_1,45_ = 40.11, *p* < 0.001); there were no significant differences among treatments (*F*_4,45_ = 1.79, *p* = 0.14; Figure 3b).

Similar to wet pectoralis, there was no effect of humidity on dry pectoralis mass (*F*_1,51_ = 0.371, *p* = 0.545), and humidity treatment was dropped from the final linear model. Pectoralis dry mass was not significantly different between post and recovery time points (*F*_1,52_ = 0.0069, *p* = 0.93; Figure 3c), after controlling for wing chord (*F*_1,52_ = 34.42, *p* < 0.001) and year (*F*_1,52_ = 4.60, *p* = 0.037). There was a significant interaction between duration in the wind tunnel and flight treatment (flight treatment x duration in wind tunnel: *F*_1,52_ = 4.48, *p* = 0.039; flight treatment: *F*_1,52_ = 1.94, *p* = 0.17; duration wind tunnel: *F*_1,52_ = 2. 86, *p* = 0.097), with flight birds losing approximately 4 mg of dry mass per hour.

The dry pectoralis mass of the preflight control birds was compared with flight and rest birds with durations in the wind tunnel greater than or equal to 5 h. Because humidity was not a significant factor in the previous model, it was not included as a factor here. Flight treatment and time point was concatenated into a single factor, and wing chord was included as a covariate. Year was also included as a factor, to account for a significant year effect on dry mass. After controlling for wing chord (*F*_1,44_ = 27.88, *p* < 0.001) and year (Year: *F*_1,44_ = 9.57, *p* = 0.0034), the concatenated flight/time point factor had a significant effect (concatenated treatment: *F*_4,44_ = 3.80, *p* = 0.010; Figure 3d). Post hoc testing revealed that the postflight birds had significantly smaller pectoralis muscles than the preflight control and post‐rest birds (24 mg, *p* < 0.02).

### 3.6 Water content

After controlling for year (*F*_1,57_ = 6.00, *p* = 0.017), there was a significant effect of an interaction between flight treatment and time point on percent water content of the muscle (flight treatment: *F*_1,57_ = 1.45, *p* = 0.233; time point: *F*_1,57_ = 0.23, *p* = 0.63; flight treatment x time point: *F*_1,57_ = 14.69, *p* < 0.001, Table 2). Post‐rest birds had lower water content compared with postflight and all of the recovery birds. Duration in the wind tunnel was also a significant factor (*F*_1,57_ = 4.95, *p* = 0.030), with longer durations in the wind tunnel associated with greater water content in the pectoralis muscle in the flight birds.

**TABLE 2:** Mean percent water content of pectoralis muscle by treatment group.

	Time point				
	Controls	Post		Recovery	
Humidity treatment		Flight	Rest	Flight	Rest
	69.3 ± 1.4				
HEWL		69.9 ± 0.8	68.6 ± 1.1*	69.7 ± 0.7	69.8 ± 1.1
LEWL		70.5 ± 1.0	69.5 ± 0.8	69.2 ± 0.8	70.2 ± 1.1

*Note*: Values are means ± SD.

* Significantly different from the other treatment groups.

### 3.7 Enzyme activity and protein content

Citrate synthase activities did not vary across any of the experimental treatment groups; flight treatment, humidity treatment, time point (post/recovery), and duration in the wind tunnel were not significant (*p* > 0.1). Because there was no consistent effect of humidity treatment and wind tunnel duration across all enzymes, these factors were dropped and we compared the control birds with postflight, post‐rest, recovery flight, and recovery rest, after combining flight treatment and time point into a single factor. There was a significant effect of treatment on mass‐specific CS activity (*F*_4,75_ = 2.5679, *p* = 0.045), with postflight recovery birds being significantly lower than preflight control birds (Tukey HSD; *p* = 0.026).

When examining mass‐specific HOAD activity in post and recovery birds, there was a significant interaction between flight treatment and time point (flight treatment: *F*_1,58_ = 0.101, *p* = 0.752; time point: *F*_1,58_ = 2.04, *p* = 0.159; flight treatment x time point: *F*_1,58_ = 4.89, *p* = 0.031), with postflight individuals having lower mass‐specific HOAD activity than post‐rest. There was no other significant factor (*p* > 0.1). After dropping humidity and duration, we compared preflight controls to the other treatment groups. Mass‐specific HOAD activity did not vary significantly with flight treatment (*F*_4,75_ = 2.43, *p* = 0.055; Figure 5b). Despite the one‐way ANOVA demonstrating no difference among all groups, post hoc analysis shows that the post‐rest birds had significantly higher mass‐specific HOAD activity compared to the postflight birds (*p* = 0.025).

There was a significant difference in mass‐specific CPT activity between years (*t*_11.66_ = −3.50, *p* = 0.0046), so year was included as a factor in subsequent models. Mass‐specific CPT activity did not vary across any of the treatment groups (*p* > 0.05). When reanalyzed with controls after dropping humidity and duration from the analysis, there were no significant differences among any of the treatment groups (*p* > 0.05).

Mass‐specific LDH activity was not significantly different among the different treatment groups (*p* > 0.05). When LDH was reanalyzed with controls after dropping humidity and duration from the analysis, there was no significant differences among any of the factors (*p* > 0.05).

There was a significant difference between 2016 and 2017 in pectoralis ALT activity in control birds (Welch's *t*‐test: *t*_7.45_ = 3.84, *p* = 0.005), so year was incorporated as a factor for all subsequent models. There was a significant difference between years for mass‐specific ALT activity (*F*_1,58_ = 6.1305, *p* = 0.0162) but no other factor was significant (*p* > 0.1). When mass‐specific ALT activity was reanalyzed with controls, after dropping humidity and duration from the analysis, there was no significant differences among treatment groups (*p* > 0.05).

**FIGURE 4:**
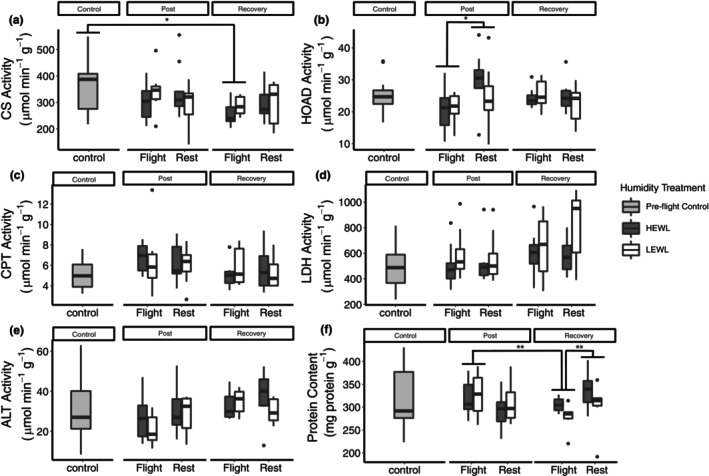
Maximum enzyme activities (V_max_) and protein content of the pectoralis major muscle from birds sampled preflight (control), immediately after exiting the wind tunnel (post) or 24 h of recovery (recovery). Humidity treatments are represented by dark gray or white for HEWL and LEWL, respectively. ALT: alanine aminotransferase; CPT, carnitine palmitoyl transferase; CS, citrate synthase; HOAD, 3‐hydroxyacyl CoA dehydrogenase; LDH, lactate dehydrogenase. * *p* < 0.05; ** *p* < 0.01. (Sample sizes of each experimental group: Flight/HEWL/Recovery = 7; Flight/LEWL/Recovery = 6; Rest/HEWL/Recovery = 7, Rest/LEWL/Recovery = 6; Flight/HEWL/Post = 9; Flight/LEWL/Post = 10; Rest/HEWL/Post = 9; Rest/LEWL/Post = 10; Preflight control = 16. The number of males, females, and unknown are 38, 39, and 3, respectively, and both sexes were represented in all experimental groups).

When protein content was assessed for only the post and recovery birds, there was a significant interaction between flight treatment and time point upon protein concentration (flight treatment: *F*_1,57_ = 12.0582, *p* = 0.0010; time point: *F*_1,57_ = 22.2474, *p* < 0.0001; flight treatment x time point: *F*_1,57_ = 16.9341, *p* = 0.0001, Figure 4f). Protein content of recovery‐flight birds was significantly lower than postflight. There was no effect of humidity or duration (*p* > 0.05). Analysis of all treatment groups, including controls, showed that there was a significant effect of treatment group on protein content (*F*_4,75_ = 5.0707, *p* = 0.0011). Post hoc testing revealed that postflight recovery was significantly lower than postflight and post‐rest recovery, by about 52.71 mg g wet tissue^−1^ and 47.31 mg g wet tissue^−1^, respectively.

### 3.8 Single fiber contractile performance

**FIGURE 5:**
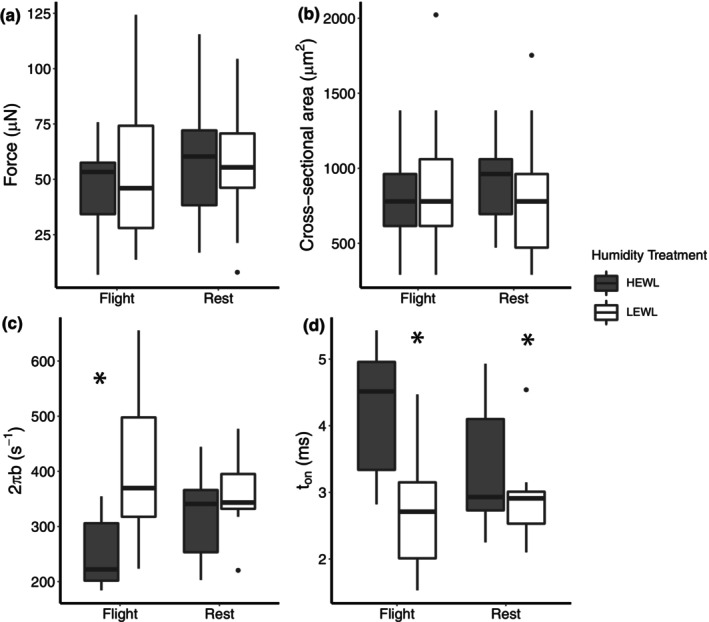
Single‐fiber contractile performance of the pectoralis muscle from yellow‐rumped warblers immediately following flight or rest treatment in HEWL and LEWL conditions. (a) Maximum force production at 25C, (b) muscle fiber cross‐sectional area, (c) apparent rate of myosin force production, and (d) mean myosin attachment time to Actin. * *p* < 0.05. There was a significant effect of humidity on mean myosin attachment time and a significant interaction between flight and humidity on 2πb.

At the fiber level, all groups showed similar force production and cross‐sectional area, indicating no significant fiber atrophy occurred during flight or humidity treatment (Figure 5a,b). Flight birds under HEWL conditions (i.e., flight‐by‐humidity interaction effects) showed slowed myosin–actin crossbridge kinetics, through a decrease in the rate of force production and a longer myosin attachment time to actin (Figure 5c,d). However, other single fiber mechanical properties, such as myofilament lattice stiffness, number of strongly bound myosin–actin heads, and crossbridge stiffness, remained unchanged during flight or humidity treatment (data not shown).

## 4 DISCUSSION

Similar to our prior studies (Gerson et al., 2020; Gerson & Guglielmo, 2011; Groom et al., 2019), fat‐free mass loss occurs both during flight and inactive fasting, but this loss of whole‐animal fat‐free mass is exacerbated under HEWL conditions. Among the flight birds, longer flights resulted in smaller pectoralis muscles. While the flights did not result in reductions in wet pectoralis mass when comparing among the 5‐h duration birds, there was a significant decline in dry pectoralis muscles in the flight group compared to the rest. This mismatch between dry and wet pectoralis mass is caused by an increase in the percent water content of the postflight birds, perhaps a result of muscle edema associated with inflammation or through changes in the osmotic balance of the muscle tissue as a result of prolonged activity. Combined with an increase in plasma creatine kinase in the flight group, both lines of evidence suggest that birds accumulated muscle damage during the experimental migratory flights. Despite this loss in pectoralis mass, however, there were few measurable changes in contractile function or oxidative capacity. There were no changes in isometric force generation, only slight changes in cross‐bridging kinetics, and minor changes in oxidative capacity. Overall, this suggests protein degradation within muscle during prolonged exercise is highly regulated, and that declines in muscle mass during flight may not be completely matched by proportional declines in muscle function.

Most significantly, there is evidence that muscle damage is taking place, as indicated by the rise in plasma creatine kinase activities and possible edema, but likely in a very controlled manner. Plasma creatine kinase has been found to be a reliable indicator of muscular damage in a variety of species (Clarkson et al., 1986), and has been documented in other bird species following a long‐distance migratory flight (Guglielmo et al., 2001). Regardless of the variability in the rest condition birds, there was still a higher amount of creatine kinase in the flight birds compared to the rest birds, indicating that flight itself was a major source of the creatine kinase. Moreover, we also saw increases in the water content of the flight birds' muscles, which may be indicative of inflammation‐induced edema. Changes in water content may be related to either changes in vasculature (vasodilation) increasing fluid content, or increases in water content of the tissues due to edema. In rats with exercise‐induced muscle damage, there can be significant increases in the total water content of the tissue (Friden et al., 1986; Komulainen et al., 1994). While increases in muscle water content and plasma creatine kinase may not provide a quantitative assessment of the severity of exercise‐induced muscle damage (Baird et al., 2012; Clarkson et al., 1986), ultrastructural changes in the muscle has previously been noted in wild birds during the migratory seasons including Z‐line streaming, loss of mitochondria, and lipofuscin bodies (George et al., 1987). These changes in muscle morphology in birds are consistent with exercise‐induced muscle damage in humans (Hikida et al., 1983).

Flapping flight in birds involves a high rate of concentric contractions (Biewener et al., 1998; Hedrick et al., 2003; Tobalske et al., 2003), which is known to cause less exercise‐induced muscle damage compared to eccentric contractions in humans (Lavender & Nosaka, 2006; Newham et al., 1986; Newham, McPhail, et al., 1983; Newham, Mills, et al., 1983). While the flight muscles may be spared from the more severe exercise damage, the repeated activation of the muscles, in excess of ~10 Hz, is the most likely source of muscle tissue damage. Work in ultramarathon runners and triathletes have found that prolonged endurance running and cycling can result in increases in markers of muscle damage during a 48‐h ultramarathon (Margeli et al., 2005). The high rate of muscular contraction compared to larger organisms may accelerate the onset of exercise‐induced muscle damage. Single fiber force production, fiber size, myofilament lattice stiffness, and number of strongly bound myosin heads remained unchanged during flight or humidity treatment, indicating these conditions did not induce significant fiber atrophy or myofilament protein loss. However, birds flown under dry air conditions showed slowing of myosin–actin crossbridge kinetics, which should decrease single fiber contractile velocity (Piazzesi et al., 2007) and could lead to lower whole muscle velocity. Notably, based upon force, size, and crossbridge kinetics, fibers from birds were most similar to myosin heavy chain (MHC) IIA muscle fibers from mice (Momb et al., 2022), indicating bird fibers contain myosin that are fast‐contracting, but not the very fast‐contracting isoforms found in MHC IIB fibers.

Dehydrating conditions impaired contractile elements, which could represent an environmental stress response to high rates of water loss, or could be directly linked to changes in water or ion homeostasis in the muscle. There is evidence of myofibrillar protein content declines following a migratory flight in garden warblers and as exercise‐induced muscle damage has a heterogenous distribution, with some muscle regions being more prone to muscle damage than others, this may help explain the limited impact humidity has on crossbridge kinetics. Given that enzyme assays were performed on relatively large masses of muscle tissue, we likely have a uniform distribution of various muscle fibers that both experienced muscle damage and that were spared from damage. However, when selecting tissue for measurement of crossbridge kinetics, fibers that appeared damaged (i.e., kinked, varied greatly in cross‐sectional area along the length of the fiber, very small diameter) were not selected to avoid biasing the results with potentially unhealthy tissue. If myofibrillar protein loss occurs in specific fibers instead of similarly across the whole muscle, then our selection criteria might not identify these fibers. Additionally, large variations in force are expected due to the large variation in fiber size (smaller fibers will produce small forces and large fibers will produce large forces). Immunohistochemistry of the entire muscle bundle would be an approach to use in future studies to determine if flight or humidity are affecting a subpopulation of skeletal muscle fibers.

While dry ambient conditions had an effect on some contractile properties, the role of ambient humidity on muscle tissue loss and degeneration was not detected, contrary to our hypothesis. Despite the impacts of HEWL on the amount of whole‐animal fat‐free mass that is lost in the animal, there were few detectable changes in muscle size or function that could be attributed to humidity exposure. This suggests one of two possible outcomes. First, the losses associated with HEWL are relatively mild for the experimental flight durations, and may not be adequately captured in our study given the potential for measurement error in excising the muscle. Second, lean mass loss induced by HEWL may not impact the flight muscles and instead target other tissues that are not be as active or essential for flight. Given that there are tissue‐specific responses to migration (Battley et al., 2000; Bauchinger et al., 2005), there may also be tissue‐specific responses to environmental stress. For example, the intestine is known to shrink the most relative to other tissue types during a migratory flight (Battley et al., 2000), and therefore may respond to a greater degree than the pectoralis to environmental stressors encountered during migration.

As muscle mass loss and whole‐animal nonfat mass loss are not directly related, the mechanisms for exercise‐ and environmentally driven protein degradation are not entirely coupled. In mammalian systems, endurance exercise causes ionic dysregulation, with Ca^2+^ becoming rapidly upregulated. This rise in Ca^2+^ may be mediated by a mismatch between ATP supply and demand during endurance exercise, reducing the ability to sequester Ca^2+^ (Tee et al., 2007). Constitutive rises in cellular Ca^2+^ may activate Ca^2+^‐sensitive proteases (calpains), which may begin the degeneration of the skeletal muscle proteins and initiate apoptosis. On the other hand, birds have a high capacity for fatty acid oxidation (Guglielmo, 2010, 2018; Guglielmo et al., 2002; McFarlan et al., 2009), supplying ATP at sufficiently high rates, sparing the muscle from damage and injury related to endurance exercise.

Glucocorticoids could be another major signal for either environmentally or exercise‐induced protein loss by mobilizing energetic stores, including amino acid catabolism. Glucocorticoids increase rates of protein degradation and decrease rates of protein synthesis in the skeletal muscle, and these free amino acids are mobilized to the liver for gluconeogenesis (Kuo et al., 2013). In migratory birds, glucocorticoids are high before departure because the birds are fasting while undergoing exercise, and then further upregulated due to high rates of metabolic expenditure during flight. Migratory birds are also primed for protein degradation due to the high circulating levels of corticosterone at the initiation of migratory departure (Eikenaar et al., 2020). This may explain the nonsignificant, but notable, rise in plasma creatine kinase in the rest condition birds. As these birds were all in the fall migratory condition, a rise in nocturnal glucocorticoid levels may have initiated protein catabolism in the flight muscle. Guglielmo et al. found a rise in plasma creatine in birds as the time between capture in the mist net and blood collection increased, suggesting a significant role of stress on skeletal muscle damage (Guglielmo et al., 2001). Birds in the rest condition may have responded to their new environment in the wind tunnel by initiating glucocorticoid‐related proteolysis and amino acid mobilization or they were already in migratory disposition and had begun mobilizing their protein stores.

The activity of autophagy and ubiquitin‐protease catabolic pathways is associated with the onset of muscle injury and recovery and rises during post‐endurance exercise recovery in mammals (He et al., 2012; Jamart et al., 2012, 2013). Moreover, autophagy in a fasted state appears to be mediated by a reduction in insulin signaling in rodents (Jamart et al., 2013). While insulin signaling in birds lacks a glucose‐regulating effect (Braun & Sweazea, 2008), insulin and insulin‐like growth factors may play an important role in regulating muscle growth and anabolism (Duclos et al., 1999; Seebacher, 2018). For example, studies on white‐crowned sparrows demonstrate that insulin‐like growth factor‐1 gene expression increases markedly predeparture compared birds in the winter condition (Pradhan et al., 2019). On the other hand, the white‐throated sparrow does not demonstrate this seasonal pattern (Price et al., 2011). Overall, given the varied mechanisms that can stimulate protein degradation, exercise and environmentally induced protein breakdown is likely mediated by a combination of these signals.

The time spent refueling and recovering from long‐duration flight is a critical determinant of migration speed and overall fitness of the individual and it seems flight muscles can completely recover in mass within as little as a few weeks in shorebirds (Gaunt et al., 1990; Piersma et al., 1999). During recovery, migratory birds show a distinct, biphasic mass gain pattern, with primarily protein and lean components recovering before the acquisition of the large fat stores (Carpenter et al., 1993; Piersma et al., 1999). As such, there is rapid gain in organ mass during the initial phase of refueling (Bauchinger et al., 2005), with digestive organs being a key priority before other organs such as the flight muscle (Bauchinger et al., 2009). However, investigations into the dynamics of recovery have relied on terminal studies and have not been done on migratory birds. Furthermore, there have been no reports about changes in physiological function during recovery.

In this study, there was evidence of rapid muscle mass recovery of flight birds following one day of recovery, with muscle mass of the recovery‐flight birds being similar to rest birds and the preflight controls. As there was not a significant difference in water content relative to control birds, this recovery of mass was not due to water influx. Recovery‐flight birds showed a significant decline in total muscle protein content relative to all other groups, suggesting that protein and amino acids were mobilized from the muscle, potentially as a metabolizable substrate or for protein synthesis of other tissues. As noted earlier, the digestive tract is one such tissue that requires rapid recovery following migration, and may rely on amino acids mobilized from the muscle to accelerate its recovery.

Delayed‐onset protein losses following a migratory flight may contribute to the time it takes for birds to recover. Reduced muscle performance could lower foraging performance and significantly hamper recovery. As demonstrated previously in garden warblers (*Sylvia borin*) (Bauchinger & Biebach, 2001), contractile proteins continue to degrade following migratory flight, and this may be the source of continued protein losses observed in the recovery‐flight animals. Research on exercised mammals has found that contractile proteins are normally spared during exercise, but are rapidly broken down during recovery (Dohm et al., 1987). In human studies on eccentric contraction, damage continues to accumulate in the days after an exercise bout (Newham, McPhail, et al., 1983), suggesting that continued losses in contractile elements may persist during the recovery following a migratory flight in birds. This corresponds to the maintenance of contractile characteristics measured here, with very little change in contractile kinetics immediately following flight activity. Moreover, *V*_max_ data from enzymes associated with ATP generation do not show any change across the different treatment groups, suggesting that energy‐producing pathways are spared during the recovery period. This is similar to a previous study on yellow‐rumped warblers that demonstrated that migratory flight in a wind tunnel does not change the enzymatic capacity (Dick & Guglielmo, 2019a), suggesting that muscle aerobic capacity does not change following flight. We do not have contractile data on recovering birds, but contractile performance may continue to be lost in the days following a migratory flight.

Recovery‐flight birds had significantly lower total protein content, but no difference in pectoralis mass, compared to the control birds. There could be significant increases in the amount of locally stored energy substrates within the muscle, such as glycogen and lipids that contribute to the apparent recovery of pectoralis mass despite an overall loss of lean mass. Considering that yellow‐rumped warblers are reported to reduce their basal metabolic rates following a migratory flight (Gerson et al., 2020), flight muscle may rapidly recover its mass as stored energy substrates (lipids and glycogen) over the course of the day rather than protein, if provided enough food.

In conclusion, migratory flight results in significant losses of skeletal tissue in yellow‐rumped warblers. Despite this damage, birds were still capable of rapidly recovering muscle tissue mass with few detectible deficiencies in muscle function. Moreover, humidity did not significantly affect the amount of muscle tissue that was lost during flight, even though it caused consistent whole‐animal differences in the rate of lean mass loss. Future work will examine the molecular and endocrinological mechanisms driving muscle loss during migration to further understand the impact of environmental stressors on phenotypic flexibility and how migratory animals have evolved to complete extraordinary feats of endurance exercise.

## AUTHOR CONTRIBUTIONS

DJEG and ARG conceived and designed the research; DJEG, ECB, JED, JGD, MCL, BPP, CRS, and KLU performed the experiments; DJEG and CRS analyzed data; DJEG, CRS, and MSM interpreted results of the experiments; DJEG prepared figures; DJEG drafted manuscript; DJEG, MSM, and ARG edited and revised the manuscript.

## FUNDING INFORMATION

National Science Foundation Grant IOS‐1656726 to ARG.

## CONFLICT OF INTEREST STATEMENT

No conflicts of interest, financial, or otherwise, are declared by the authors.

## ETHICS STATEMENT

All animal procedures and care followed Canadian Council on Animal Care guidelines and were approved by Western University Animal Care Committee (protocol 2010‐216) and the University of Massachusetts Amherst Institutional Animal Care and Usage Committee (protocol 2015‐0019). Permission for animal capture was provided by the Canadian Wildlife Service (permit CA‐0256 to Dr. Christopher Guglielmo).

## Data Availability

Data are available through FigShare: https://doi.org/10.6084/m9.figshare.22207420.v1
